# Growth Factor Binding Peptides in Poly (Ethylene Glycol) Diacrylate (PEGDA)-Based Hydrogels for an Improved Healing Response of Human Dermal Fibroblasts

**DOI:** 10.3390/gels9010028

**Published:** 2022-12-29

**Authors:** Abigail J. Clevenger, Andrea C. Jimenez-Vergara, Erin H. Tsai, Gabriel de Barros Righes, Ana M. Díaz-Lasprilla, Gustavo E. Ramírez-Caballero, Dany J. Munoz-Pinto

**Affiliations:** 1 Engineering Science Department, Trinity University, San Antonio, TX 78212, USA; 2 Chemistry Department, Trinity University, San Antonio, TX 78212, USA; 3 Neuroscience Program, Trinity University, San Antonio, TX 78212, USA

**Keywords:** human dermal fibroblasts, growth factors, hydrogels, binding peptides

## Abstract

Growth factors (GF) are critical cytokines in wound healing. However, the direct delivery of these biochemical cues into a wound site significantly increases the cost of wound dressings and can lead to a strong immunological response due to the introduction of a foreign source of GFs. To overcome this challenge, we designed a poly(ethylene glycol) diacrylate (PEGDA) hydrogel with the potential capacity to sequester autologous GFs directly from the wound site. We demonstrated that synthetic peptide sequences covalently tethered to PEGDA hydrogels physically retained human transforming growth factor beta 1 (hTGFβ1) and human vascular endothelial growth factor (hVEGF) at 3.2 and 0.6 ng/mm^2^, respectively. In addition, we demonstrated that retained hTGFβ1 and hVEGF enhanced human dermal fibroblasts (HDFa) average cell surface area and proliferation, respectively, and that exposure to both GFs resulted in up to 1.9-fold higher fraction of area covered relative to the control. After five days in culture, relative to the control surface, non-covalently bound hTGFβ1 significantly increased the expression of collagen type I and hTGFβ1 and downregulated vimentin and matrix metalloproteinase 1 expression. Cumulatively, the response of HDFa to hTGFβ1 aligns well with the expected response of fibroblasts during the early stages of wound healing.

## 1. Introduction

Currently, the standard therapies to treat burns, skin lesions, and chronic and diabetic wounds include the control of infections, acute inflammation, debridement of the wound site, and the use of appropriate dressings [1,2,3,4,5]. However, these techniques are only efficient if the vascularization of the wound is not impaired. Therefore, research efforts have been focused on the fabrication of bioengineered skin substitutes with the potential to deliver cells or proteins to aid in re-vascularization. These types of wound dressings have shown promising results and some of them are currently commercially available [6]. Specifically, wound dressings containing growth factors (GFs) such as transforming growth factor beta 1 (TGFβ1), vascular endothelial growth factor (VEGF), and epidermal growth factor (EGF) have promoted healing by stimulating cell growth, proliferation, differentiation, and the development of new blood vessels [6,7,8,9]. Despite the advantages of these types of dressings, they have limited use due to low availability, high cost, and the risk of a strong immunological response by the introduction of a foreign source of GFs [10]. Consequently, the development of an effective wound-healing strategy requires the use of affordable materials that can deliver growth factors while minimizing undesired immune responses [7,11]. Thus, the main goal of this exploratory work was to develop and evaluate an in vitro family of biomaterials for the fabrication of wound dressings with the capacity to capture endogenous GFs. The proposed dressings have the potential to sequester and retain GFs directly from the wound site and create a GF-enriched surface that promotes healing from adjacent healthy tissue. By advantageously recruiting and retaining GFs from the wound site, it is no longer necessary to introduce GFs from exogenous sources. The proposed biomaterials concentrate the GFs directly at the wound site, where they are most essential, and since they do not incorporate extrinsic sources of GFs, the likelihood of biological incompatibility is reduced.

This study was focused on the preliminary in vitro evaluation of a family of poly(ethylene glycol) diacrylate (PEGDA) based hydrogels that incorporate synthetic peptide sequences with the capacity to specifically bind to selected GFs as potential wound dressings. The modification of polymer-based surfaces has been used to enhance the properties of biomaterials to exhibit responses to changes in temperature or pH, to regulate antimicrobial activity, and to modulate cellular responses such as cell attachment [12]. Enhancing the properties of PEG-based hydrogels with affinity peptides is a promising strategy to deliver specific biomolecules [13]. By employing synthetic GF binding peptide sequences, we engineered PEGDA-based dressings with the capacity to sequester human TGFβ1 (hTGFβ1) and human VEGF (hVEGF). The use of a synthetic binding peptide instead of foreign GFs will significantly reduce the dressing cost and improve its commercial availability. The GFs, hTGFβ1 and hVEGF, were selected as targets in our strategy due to their essential role in wound healing and tissue repair. It is established that the release of TGFβ1 stimulates the migration of fibroblasts toward the wound site and initiates the synthesis of collagen, glycoproteins, glycosaminoglycans, tropoelastin, and elastin fibers, which are critical components responsible for rebuilding damaged extracellular matrix (ECM) tissue [14,15]. Additionally, VEGF released by macrophages initiates angiogenesis to supply the growing tissue with adequate nutrients and oxygen while also directing the migration of inflammatory cells to the site of injury [10,16]. PEGDA was selected as a base material for our hydrogel system due to its biocompatibility, pro-healing properties (i.e., maintaining a moist environment), thermal stability, and low-adherent properties which facilitate easy, non-destructive removal of the material from the wound site [17]. Hydrogels composed of synthetic polymers such as PEGDA can donate necessary moisture to dry wounds, reduce the wound bed temperature up to 5 degrees Celsius, and, most importantly, increase granulation and epithelialization [18]. In addition, PEGDA hydrogels can be easily fabricated in aseptic conditions using a light source and are designed to contain and deliver water-based antiseptic and topical medications. Lastly, the composition of PEGDA-based hydrogel surfaces can be conditioned to physically recruit and retain GFs through non-covalent bonding. Therefore, we hypothesize that the developed dressing can physically bind and retain the naturally produced hTGFβ1 and hVEGF within the wound site. This approach will simultaneously preserve endogenous GF bioactivity and enable continuous GF localization on the surface of the dressing, which are two variables needed to enhance the overall healing rate in the wound area.

**Figure 1:**
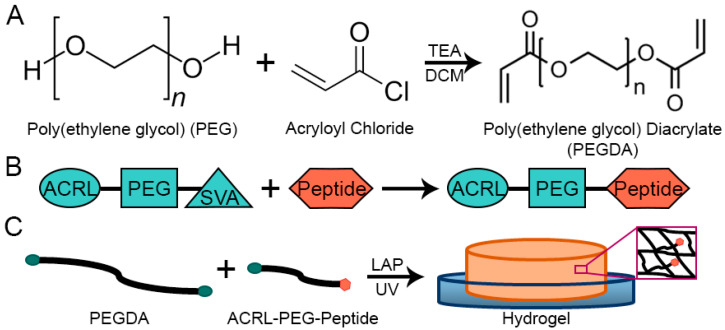
(**A**) Schematic representation of the end-group modification of PEG. (**B**) Schematic representation of the conjugation of peptide sequences to a PEG-based linker, and (**C**) illustration of the incorporation of conjugated peptides into a PEGDA-based hydrogel using photopolymerization.

To fabricate our proposed system, the terminal hydroxyl groups in linear PEG chains were replaced by acrylate groups. GF binding peptide sequences selected based on previously reported research by J. Crispim et al. [19] and A. Adini et al. [20] were conjugated with an acrylate-terminated PEG linker. The conjugated peptide sequence was then covalently incorporated to the PEGDA hydrogel using UV polymerization (Figure 1). The successful incorporation of the binding peptide into the PEGDA-based hydrogel was confirmed using Attenuated Total Reflection Fourier Transform Infrared Spectroscopy (AT-FTIR). The capacity of the proposed hydrogels to sequester and physically retain hTGFβ1 and hVEGF was also quantitatively evaluated. Following the successful confirmation of the retention of human GFs in the engineered dressings, we evaluated their biological activity and effects on human dermal fibroblasts (HDFa) under in vitro culture conditions. Fibroblasts were selected due to the primary role that they play in the healing process in open wounds. The biological responses of HDFa to the physically bound hTGFβ1 or hVEGF on PEGDA-based hydrogels were evaluated in terms of cell proliferation, average cell surface area, cell surface coverage, and phenotypical expression at the gene level. The cumulative set of results demonstrated that the use of the proposed dressings containing physically bound GFs increased the proliferation of HDFa, average cell surface area, and the total area covered by the cells. In addition, in response to the presence of hTGFβ1 and hVEGF on the hydrogels, HDFa exhibited an increase in the expression of collagen type I (ColI) and TFGβ1 and a decrease in the expression of vimentin and matrix metalloproteinase 1 (MMP1). Collectively, our results indicate that our proposed dressing properties are a promising avenue for future wound care applications.

## 2. Results

### 2.1. Peptide Functionalization and Incorporation into PEGDA-Based Hydrogels

**Figure 2:**
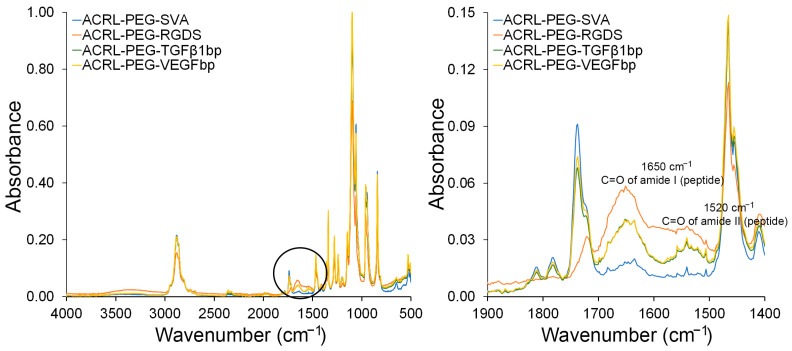
ATR-FTIR spectra of ACRL-PEG-SVA (blue), ACRL-PEG-RGDS (orange), ACRL-PEG-TGF𝛽1bp (green), and ACRL-PEG-VEGFbp (yellow). ATR-FTIR results confirmed the conjugation of the peptide binding sequences to ACRL-PEG.

PEGDA-based hydrogel dressings have been successfully used to treat wounds [21,22]. They provide an initial physical barrier, can be removed easily without increasing wound damage, and have the capacity to retain moisture and deliver target-specific molecules including antibiotics and growth factors [23,24,25,26]. In our approach, we increased the complexity of PEGDA-based dressings by modifying the composition of PEGDA-based hydrogels by the introduction of selected chemical motifs. In this way, we enhanced the chemical functionally of the otherwise blank slate of the PEGDA network. Toward this end, the binding peptide sequences KGLPLGNSH (TGFβ1bp) and DRVQRQTTTVVA (VEGFbp) were functionalized using ACRL-PEG-SVA. The SVA ester group allowed for the covalent conjugation to the N-terminal in the peptide sequence while the acrylate group permitted the anchoring of the selected peptides into the structure of the hydrogel during the UV polymerization. The successful conjugation of the peptide sequences to the ACRL-PEG-SVA linker was qualitatively confirmed using ATR-FTIR (Figure 2).

Some of the characteristic infrared absorption bands for PEG acrylate-based polymers and peptide structures are summarized in Table 1 [27,28]. Across all samples, the PEG signature is present and primarily confirmed by the absorption bands at 2889 cm^−1^ and 1110 cm^−1^. These bands are maintained across all species, the control ACRL-PEG-SVA, and the peptide containing polymer chains. 

Relative to the pure ACRL-PEG-SVA linker, the ATR-FTIR spectra showed an increase in the absorption peaks at 1650 cm^−1^ and 1520 cm^−1^ corresponding to the amide I and amide II vibration bands of the C=O bond in the amide group in the samples containing the peptide sequences. The relative ratio between the absorption bands at 1650 cm^−1^ and 2889 cm^−1^ (CH_2_ stretching) in the PEG chain [29] also suggested that the RGDS sequences had higher functionalization levels while the TGFβ1bp and VEGFbp sequences appeared to be functionalized to a similar extent.

### 2.2. Physical Characterization

The designed PEGDA-based hydrogels were characterized in terms of their mechanical performance and swelling behavior. The average complex modulus, stiffness, and swelling ratios at equilibrium after swelling and in the relaxed state are reported in Table 2. The swelling data were used to estimate the average molecular weight between crosslinks (Mc = 17.5 ± 1.0 kDa) and the average mesh size (ξ = 21.9 ± 1.0 nm) in the hydrogel systems [30]. The resulting hydrogels exhibited high water retention capacity at approximately 15.5 times the mass of the dry polymer. In aqueous environments, the open mesh structure (approximately 22 nm) of these PEGDA-based hydrogels allowed for the diffusion of large molecules including polymers such as dextran and proteins with MWs up to 67,000 g/mol [30,31]. The high water uptake and mesh diffusional properties are highly desirable in wound dressings because water-soluble antibiotics of low molecular weights, such as penicillin, tetracycline, ciprofloxacin, and moxifloxacin, among others, may be delivered using the proposed dressings [32]. A dressing with the capacity to retain and deliver antibiotics is critical in preventing and treating a bacterial infection in a wound site [4]. 

### 2.3. Growth Factor Binding and Retention Capacity on PEGDA-Based Hydrogels

The bound levels of hTGFβ1 or hVEGF on the corresponding PEGDA-peptide hydrogels were quantified using the CBQCA assay. As shown in Table 3, both the PEGDA-TGFβ1bp and the PEGDA-VEGFbp hydrogels were able to sequester their corresponding GF after 1 h of contact time with the hydrogel surface. The 1 h contact time was selected to evaluate the capacity of the external hydrogel surface to retain the GFs and limit the effects of their absorption and retention by diffusion into the bulk structure of the hydrogels. Bulk assessments of absorption of protein levels required at least 24 h contact time [33]. The results presented and summarized in Table 3 show the net amount of bound GF after subtracting the background reading from the corresponding PEGDA control group. CBQCA results indicated that PEGDA-TGFβ1bp exhibited a binding capacity of 3.19 ± 0.34 ng/mm^2^ while PEGDA-VEGFbp displayed a binding capacity of 0.62 ± 0.06 ng/mm^2^. Based on these results, we were able to confirm that the network of the PEGDA-peptide hydrogels had the capacity to capture and physically retain hTGFβ1 and hVEGF.

### 2.4. Bioactivity of Growth Factors Bound to PEGDA-Peptide Hydrogels

Growth factors (GFs) such as TGFβ1 and VEGF are common additions to wound dressings for their promotion of healing through cell stimulation, proliferation, differentiation, and angiogenesis [34,35]. The therapeutic potential of the developed PEGDA-peptide hydrogels as wound dressings for dermis regeneration was evaluated under in vitro conditions using adult human dermal fibroblasts (HDFa). The biological responses of HDFa to physically bound hTGFβ1 or hVEGF were evaluated in terms of cell proliferation and gene expression.

**Figure 3:**
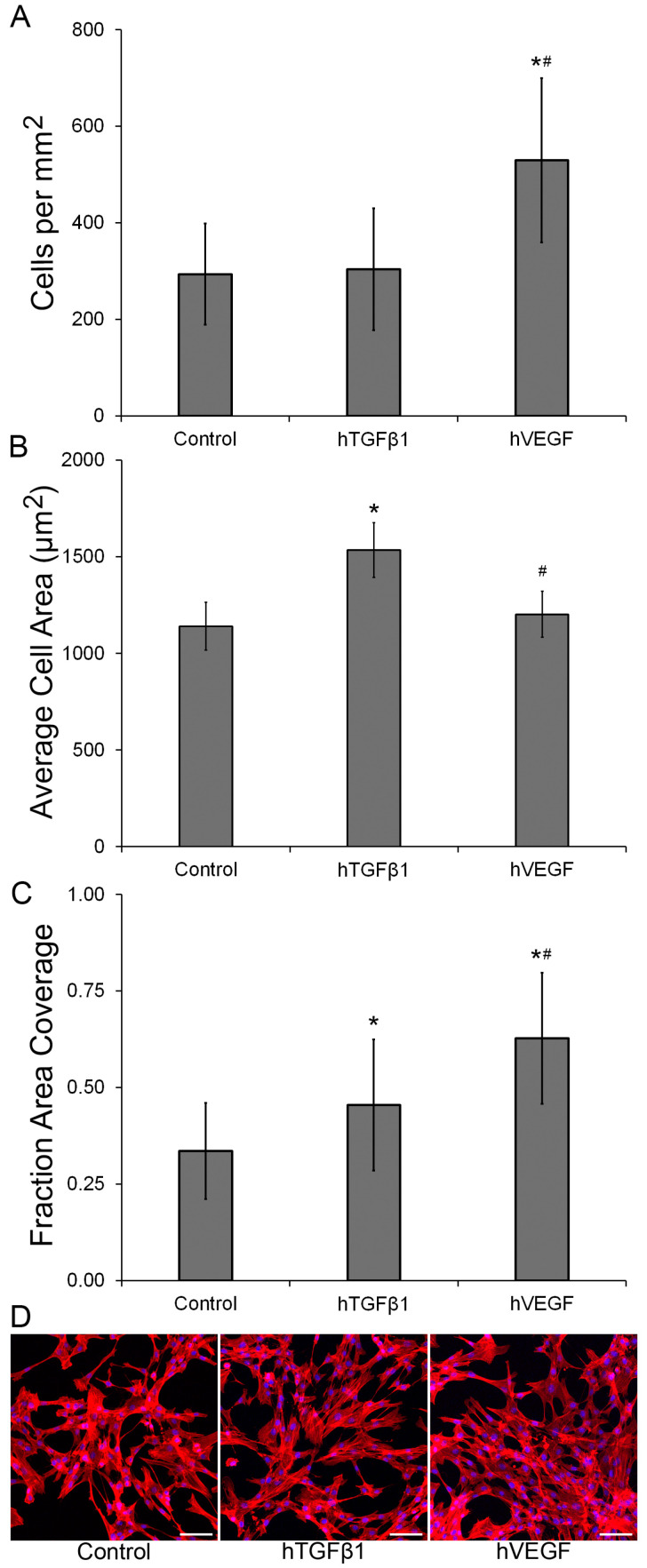
(**A**) Total number of cells per area after 3 days of culture, (**B**) Average cell area, (**C**) Fraction of area covered by HDFa after 3 days of culture, and (**D**) Representative images of HDFa stained with Rhodamine Phalloidin (Red) and DAPI (Blue). The scale bar represents a distance of 100 µm. * Significantly different from control, *p* < 0.05, # Significantly different from hTGFβ1, *p* < 0.05. A total of 29 to 36 images were taken from 3 independent specimens per formulation.

HDFa seeded on PEGDA-peptide hydrogels containing hVEGF showed a significant increase in cell proliferation (*p* < 0.0001) relative to hydrogels containing hTGFβ1 and control (Figure 3A,D). In addition, the average cell surface area (Figure 3B) was approximately 1.3-fold higher in the hTGFβ1 group when compared to either the control or the hVEGF-containing surface (*p* < 0.001). Interestingly, the fraction of area covered by HDFa (Figure 3C) was enhanced in the PEGDA-peptide hydrogels relative to the control (*p* ≤ 0.004). The fraction of area covered was 1.9-fold higher in the hVEGF hydrogel and 1.4-fold higher in the hTGFβ1 sample relative to the control surface. Relative to the control group, it appears that the fraction of surface covered was increased in the two GF-containing hydrogels due to two different factors. In the hTGFβ1 group, the fraction of area covered was increased due to higher average cell size while in the hVEGF surface, the fraction of area covered was higher due to higher cell surface density.

**Figure 4:**
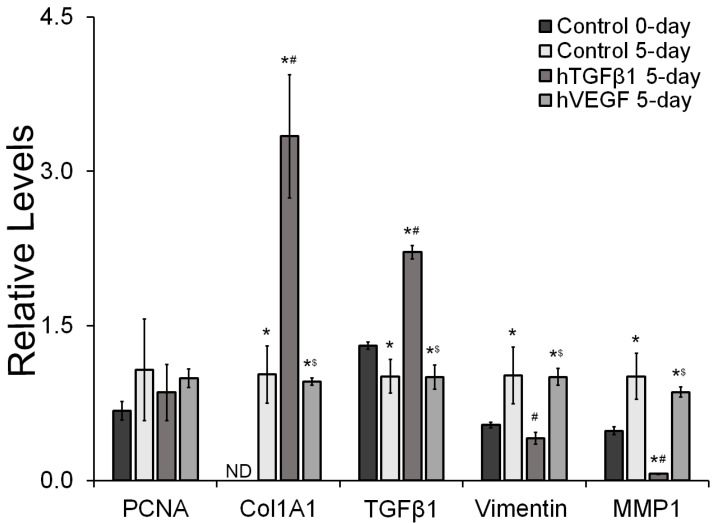
Phenotypical evaluation of HDFa using qRT-PCR. The expression of each marker was normalized relative to the 5-day control group. * Significantly different from 0-day control, *p* < 0.05, # Significantly different from 5-day control, *p* < 0.05, $ Significantly different from hTGFβ1, *p* < 0.05. ND, below the detection limit. *n* = 4.

To further evaluate the biological responses of HDFa to physically bound hTGFβ1 and hVEGF, the expression of proliferative, fibrotic and fibroblast makers were evaluated at the gene expression level (Figure 4). The qRT-PCR results showed no significant differences in the expression of the proliferation marker PCNA (*p* ≥ 0.387) across all groups. The PEGDA-peptide hydrogel containing hTGFβ1 induced a significant increase in the expression of the fibrotic markers Col1A1 and TGFβ1 (*p* ≤ 0.001) relative to all other experimental groups. The expression of Col1A1 was below the detection limit in HDFa seeded on the 48-well plates (0-day control). Col1A1 expression was 3.3-fold and 3.5-fold higher in the hTGFβ1 containing scaffold relative to 5-day control and the hVEGF-containing surface, respectively, and no significant differences in expression of Col1A1 were observed between the 5-day control group and cells exposed to hVEGF (*p* = 0.975). Relative to the 0-day control group, the expression of TGFβ1 was significantly reduced by 1.3-fold in the 5-day control and in the hVEGF group (*p* ≤ 0.041). Furthermore, relative to all other experimental groups, the expression of TGFβ1 was significantly enhanced (*p* < 0.001) by the presence of physically bound hTGFβ1 on the surface of the hydrogels. Relative to the 0-day and 5-day controls, and the hVEGF group, the expression of TGFβ1 was 1.7-fold, 2.2-fold, and 2.2-fold higher, respectively. In addition, the expression of the fibroblast marker vimentin was very dynamic across the different experimental groups. Relative to the 0-day group, the expression of vimentin was significantly upregulated by approximately 1.9-fold in the 5-day control and the hVEGF group (*p* ≤ 0.019). Cells exposed to physically bound hTGFβ1 exhibited lower expression of vimentin by 2.5-fold and 2.4-fold relative to the 5-day, and hVEGF group, respectively (*p* ≤ 0.005). Significant differences in vimentin expression were not observed between the hTGFβ1 group and the 0-day control group (*p* = 0.723). The expression profile of MMP1, another fibroblast marker, was similar to that observed in vimentin. MMP1 expression increased in the 5-day control and the hVEGF group when compared with the 0-day control by 2.1-fold and 1.8-fold, respectively (*p* ≤ 0.017). Moreover, the expression of MMP1 was significantly reduced in the hTGFβ1 group by 7.6-fold, 16.0-fold and 13.6-fold relative to the 0-day control, 5-day control and the hVEGF group, respectively (*p* ≤ 0.009).

## 3. Discussion

Ideal wound dressing features include moisture control, infection prevention, gas permeability, mechanical stability, biocompatibility, biodegradation, and low adherence to skin, among others [36]. PEG-based hydrogels can be designed to display these desirable characteristics as they naturally retain large quantities of water and are permeable to gas exchange. The mechanical performance and mesh size structure of PEG-based hydrogels are easily modulated within a broad range of values by changes in molecular weight, polymer concentration, and secondary cross-linkable molecules of varying functionality and multi-arm structures [30,37]. Specifically, the elastic modulus of PEG-based hydrogels can be adjusted to target similar properties as those present in skin structures. Our DMA results indicated that our proposed hydrogels exhibited an average complex modulus of 47.7 kPa and average stiffness of 1.9 kN/m. Previously reported modulus values for skin-based structures fall between 5 kPa and 140 MPa demonstrating that our hydrogels fit well within the expected range for elastic properties [38,39]. However, our stiffness values are between 6-fold and 4-fold higher than previously reported hydrogel results using a DMA [40]. The variability in reported mechanical properties data is due to differences in the mechanical performance of skin tissue derived from different sample locations and different testing methodologies and conditions. For our purposes, chronic, or diabetic, wounds have increased stiffness values compared to controls lending our hydrogel to remain applicable to chronic wound care. Further, fine-tuning the mechanical performance of our hydrogel can be achieved to mimic different skin locations by modulating the concentration of PEGDA and the polymer chain length and adding small cross-linkable species [41,42,43]. The diffusional properties can also be tuned to retain and deliver water-soluble therapeutic agents including antibiotics and biomolecules to regulate healing. In addition, PEG-based hydrogels can be designed to exhibit degradable motifs such as ester-based sequences which are susceptible to hydrolysis. Moreover, peptide sequences susceptible to matrix metalloprotease (MMP) degradation could also be incorporated to modulate the degradation rate of the scaffolds. Therefore, different strategies could be employed to modulate the diffusional properties of PEG-based dressings and their drug delivery potential. Recent efforts in wound healing and drug delivery have been focused on the characterization of new materials to control the delivery of antibiotics and analgesics [3,44]. This research direction could be further explored in the future using the proposed platform. 

In this exploratory study, we used PEGDA to evaluate the effects of hVEGF and hTGFβ1 physically bound to two different binding peptide sequences through the response of human dermal fibroblasts (HDFa) proliferation and phenotype modulation independently of matrix degradation, mechanical performance and diffusional properties. PEGDA hydrogels were selected as our matrix due to their well know biocompatibility, physical and chemical stability, and biological blank slate performance [23,45,46]. We focused our attention on the evaluation of hVEGF and hTGFβ1 as they are critical to the wound healing process [47,48]. Exogenous sources of VEGF and TGFβ1 have been extensively used as active components of biomaterials to promote cell proliferation, cell migration, differentiation, and the formation of new vascular structures in damaged tissue [48,49]. In most tissue regeneration strategies, exogenous GFs are released from biomaterials by simple diffusion. In other approaches, GFs are chemically modified and covalently linked to engineered scaffolds, which in most cases results in the reduction in the bioactivity of the GFs [50,51]. Therefore, the use of exogenous sources of GFs may hinder tissue regeneration in a wound site due to adverse effects such as the uncontrolled and undesired release of GFs on healthy surrounding tissue and inflammatory response from the host tissue [51].

To address this challenge, we covalently linked synthetic peptide sequences to PEGDA-based hydrogels and demonstrated that the sequences retained their capacity to physically bind to hTGFβ1 and hVEGF. The percent of retained GFs on the PEGDA hydrogels was higher in the hTGFβ1 system (42.80%) than in the hVEGF-peptide pair (9.38%). These results indicate higher affinity between the peptide and its corresponding GF for the hTGFβ1 system than for the hVEGF-peptide complex. The specific surface retention levels of GFs are in the same order of magnitude as previously reported values for hTGFβ1 physically bound to polycaprolactone-based films [19]. Differences in the absolute value between our measurements and previously reported values in literature may be derived from different experimental conditions such as the permeability of the scaffolds and testing methodologies. Using synthetic peptides to physically sequester, retain and deliver endogenous GFs has been a successful strategy to promote the regeneration of ligament, tendon and bone tissue [19,52]. Using synthetic peptide sequences has also recently been employed in the design of new wound dressings for skin tissue repair. However, the use of this strategy is still understudied in advancing chronic wound care [53].

This exploratory research lends itself as a proof of concept for the potential use of different synthetic peptide sequences to physically sequester GFs for cutaneous repair. In this work, we investigated the retention of the bioactivity of physically bound hTGFβ1 and hVEGF using HDFa as our cell model. Dermal fibroblasts were selected as our target cell due to their high abundance in cutaneous tissue and the key role they play during wound healing. These cells are responsible for the production and remodeling of ECM proteins and the regulation of the migration of inflammatory cells into the wound site and different inflammatory stages [54]. Cumulatively, our results indicate that physically bound hTGFβ1 and hVEGF on PEGDA hydrogels retained their activity. The presence of these GFs on the hydrogel surface resulted in significant changes in cell behavior. We observed a significant increase in the percentage of area covered for both GFs relative to the control group, a significant increase in total cell number on the surface treated with hVEGF, and an increase in the average cell size in the hTGFβ1 group for the time frame of the experiment. Under the tested conditions, the presence of hVEGF appears to be more effective at enhancing HDFa proliferation than the presence of hTGFβ1, especially considering that hVEGF levels on the hydrogels were lower than the hTGFβ1 surface content. Our results agree with previously reported fibroblast proliferation observations due to the use of VEGF [55,56]. Furthermore, the presence of hTGFβ1 showed a significant impact on increasing the average cell area of HDFa. The average cell size varied between 1140 μm^2^ and 1535 μm^2^. These values in average cell size are within the range of values previously reported for fully spread HDFa [57]. The presence of hTGFβ1 also modulated the cell surface area. Significantly higher average cell size in this group was observed relative to the control or hVEGF-containing surfaces. The modulation of cell morphology, including cell size and length, has been previously documented with the use of TGFβ [58]. An increase in cell surface area is a desirable response from dermal fibroblasts which contributes to healing and wound closure. 

In addition to changes in cell proliferation, cell size and changes in the fraction of area covered, we evaluated the expression of fibroblast markers including collagen type I, TGFβ1, vimentin and MMP1. Overall, at the gene expression level, the exposure of HDFa to hTGFβ1 resulted in a promising cellular response as the expression of collagen type I and TGFβ1 was increased relative to the other groups. An increase in collagen I is expected as damaged tissue regenerates and deposits new ECM and the production of TGBβ1 promotes and regulates the initial healing response of HDFa in a wound site. Moreover, relative to the day zero control group, the expression of vimentin was not significantly modified by the presence of hTGFβ1 and it was downregulated relative to the 5-day control group and the hVEGF surface. This response is desirable since an increase in vimentin expression has been linked to aberrant cell behavior and fibroblast aging [59]. In terms of MMP1 expression, the presence of hTGFβ1 significantly reduced its expression in HDFa relative to all other experimental groups. Cumulatively, our data indicate that for the time frame of our experiments, the surface containing physically retained hTGFβ1 led to the most positive HDFa response across groups. This response was characterized by the upregulation in collagen type I and TGFβ1 expression, a reduction in vimentin expression, and the increase in average cell size that has been previously linked to a decrease in MMP1 expression [60].

## 4. Conclusions

In this exploratory work, we evaluated the use of synthetic peptides with the capacity to physically retain hTGFβ1 and hVEGF. We confirmed and established the retention levels of these growth factors (GFs) on the surface of PEGDA-based hydrogels. The biological activity of these GFs was also confirmed by changes in cell proliferation and average cell surface area of human dermal fibroblasts (HDFa). In addition, changes in the expression of collagen type I, TGFβ1, vimentin, and MMP1 expression across experimental groups were confirmed at the gene expression level. The exposure of HDFa to hVEGF led to an increase in the total fraction of cell coverage due to an increase in cell proliferation. Cumulatively, the resulting response of HDFa was most significant when exposed to physically retained hTGFβ1. After 5 days in culture, HDFa exposed to hTGFβ1 showed a higher fraction of surface covered, higher expression of collagen type I and TGFβ1, and a reduction in expression of vimentin and MMP1. This response aligns well with the expected response of fibroblasts during the early stages of wound healing. Future directions will focus on the study of the synergistic effects of the simultaneous exposure of HDFa to both GFs, the exploration of hydrogel-based dressings fabricated using 3D printing technologies to introduce microtextural features on the hydrogels, and the potential evaluation of these scaffolds in a rodent-based model.

## 5. Materials and Methods

### 5.1. Synthesis of PEGDA and Photoinitiator

Polyethylene glycol diacrylate (PEGDA) was synthesized from commercially available 6.0 kDa linear PEG (Sigma-Aldrich, St. Louis, MO, USA) as previously described [30]. In short, dried PEG was combined with acryloyl chloride (Sigma-Aldrich, molar ratio 4:1) in anhydrous dichloromethane (Sigma-Aldrich). Triethylamine (Sigma-Aldrich, molar ratio 2:1) was slowly added and the resulting solution was left to react at 4 °C for 12 h. The reaction product was purified and dried under vacuum. The PEGDA acrylation level (≈97%) and degree of polymerization (DP ≈ 141) was confirmed by ^1^H NMR using a Varian 500 MHz NMR spectrometer.

Lithium phenyl-2,4,6-trimethylbenzoylphosphinate (LAP) photoinitiator was synthesized according to the protocol described in Fairbanks et al. [61]. In brief, 3 g of dimethyl phenylphosphine (Alfa Aesar, Tewksbury, MA, USA) was combined with 3 g of 2,4,6-trimethylbenzoyl chloride (Alfa Aesar) under an inert nitrogen atmosphere. After 18 h of stirring, lithium bromide (6.1 g, Acros Organics, Morris Plains, NJ, USA) was dissolved in 2-butanone (100 mL, Fisher Chemical, Whippany, NJ, USA) and heated at 50 °C. After 10 min, a solid precipitate was formed and the mixture was allowed to rest for 4 h. After purification and drying under vacuum, LAP was characterized by ^1^H NMR. A working solution of LAP (1 mM) was prepared in water.

### 5.2. Synthesis of Acrylate-Derived GF Binding Peptides and RGDS

The GF binding peptides, KGLPLGNSH [19] (Genscript, Piscataway, NJ, USA), targeting the human Transforming Growth Factor beta 1 (hTGFβ1), and DRVQRQTTTVVA [20] (Genscript), targeting the human Vascular Endothelial Growth Factor (hVEGF), and the cell adhesion peptide, RGDS (Genscript), were conjugated to Acrylate-PEG-Succinimidyl Valerate (ACRL-PEG-SVA 3.4 kDa, Laysan Bio, Arab, AL, USA). The peptides were dissolved in a 50 mM NaHCO3 pH 8.5 buffer and reacted with ACRL-PEG-SVA at a 1:1 molar ratio for 2 h. The products (ACRL-PEG-KGLPLGNSH, ACRL-PEG-DRVQRQTTTVVA and ACRL-PEG-RGDS) were purified through a dialysis membrane (3.5 kDa, Thermo Fisher Scientific, Waltham, MA, USA) for 24 h and then lyophilized. The products were stored at −20 °C until further use. Peptide conjugation to ACRL-PEG-SVA was confirmed using ATR-FTIR.

### 5.3. Fabrication of PEGDA-Based Hydrogels

Hydrogel precursor solutions were prepared by dissolving PEGDA (10% *w*/*w*), 1 mM ACRL-PEG-RGDS and ACRL-PEG-KGLPLGNSH or ACRL-PEG-DRVQRQTTTVVA at 1 mM concentration in Dulbecco’s Phosphate-Buffered Saline (DPBS, Corning, VA, USA) as shown in Table 4. The photoinitiator, 10 μL of 100 mM LAP solution, was added per 1 mL of hydrogel precursor solution. The solution was sterilized by filtration using a 0.22 µm filter (EMD Millipore) and poured into 0.75 mm thick transparent rectangular molds. The precursor solution was then polymerized by exposure to UV light (Spectroline, Melville, NY, USA, ≈6 mW/cm^2^, 365 nm) for 5 min. Each hydrogel was transferred to a Petri dish (Santa Cruz Biotechnology, Inc., Dallas, TX, USA), rinsed twice with DPBS and stored in DPBS at 4 °C for 24 h. A negative control hydrogel was prepared without incorporating either peptide binding sequence.

### 5.4. Mechanical and Swelling Characterization

The complex modulus and the stiffness of the proposed hydrogels were measured as described by Jimenez et al. [62] using dynamic mechanical analysis (DMA). In brief, four independent disks, approximately 8 mm in diameter, were cored from 1.1 mm thick hydrogel slabs. Samples were exposed to an oscillatory wave of 100 μm in amplitude and 1 Hz of the frequency following an initial preload of approximately 2 g. The complex modulus (E*) was calculated as:

(1) $$E^{*}=\sqrt{E^{\prime 2}+E^{\prime \prime 2}}$$

where *E*′ and *E*″ are the storage and loss modulus, respectively.

To estimate the average mesh size (ξ) of the hydrogels, the average molecular weight between crosslinks (M_c_) was calculated using mass swelling data and a semi-empirical correlation for PEGDA-based systems (Equation (10)) previously reported by Jimenez et al. [30]. In brief, approximately 200 μL of polymer precursor solution was placed per well in a 48-well plate (*n* = 4) and polymerized as described in Section 5.3. The initial mass (relaxed mass, m_i_) of each construct was recorded. The constructs were then transferred and allowed to swell for 24 h in DPBS. Following the incubation time, the mass of the swollen scaffolds (m_s_) was measured. After swelling, the specimens were allowed to dehydrate at room temperate for 12 h and then transferred to a lyophilizer for 24 h. The dry mass (m_d_) of each specimen was then recorded. The ξ was then calculated as

(2) ξ=ν2,s−13(r¯02)12

where $\nu_{2,s}$ is the polymer volume fractions after equilibrium and (r¯02)12 is the end-to-end distance of the unperturbed (solvent-free) state of the polymer which can be computed by the relation

(3) (r¯02)12=l(2McMr)12Cn12

where *l* is the weighted average bond length (1.50 Å), *M_r_* is the molecular weight of the repeating unit (44 g/mol), and *C_n_* is the characteristic ratio of the polymer (4 for PEG). Using Equations (4)–(10), *M_c_* was calculated.

(4) $$q=\frac{m_{s}}{m_{d}}$$

(5) $$q^{\prime }=\frac{m_{i}}{m_{d}}$$

(6) $$Q=1+\frac{\rho_{p}}{\rho_{s}}\left(q-1\right)$$

(7) $$Q^{\prime }=1+\frac{\rho_{p}}{\rho_{s}}\left(q^{\prime }-1\right)$$

(8) $$\nu_{2,s}=\frac{1}{Q}$$

(9) $$\nu_{2,r}=\frac{1}{Q^{\prime }}$$

(10) $$\frac{1}{M_{c}}=-0.109\left(\frac{\nu_{2,r}}{\nu_{2,s}}\right)\frac{\left(\frac{\bar{\nu }}{V_{1}}\right)\left[\ln\left(1-\nu_{2,s}\right)+\nu_{2,s}+{\chi \nu }_{2,s}{}^{2}\right]}{\nu_{2,r}\left[{\left(\frac{\nu_{2,s}}{\nu_{2,r}}\right)}^{\frac{1}{3}}-\frac{1}{2}\left(\frac{\nu_{2,s}}{\nu_{2,r}}\right)\right]}$$

where *ρ*_p_ and *ρ*_s_ are the densities of the polymer and the solvent respectively, $\bar{\nu }$ is the specific volume of the polymer (0.893 cm^3^/g for PEG), V_1_ is the molar volume of the solvent (18 cm^3^/mol) and χ is the polymer-solvent interaction parameter (0.426 for PEG-water systems) [63].

### 5.5. Quantification of PEGDA-Peptide Hydrogel Binding Capacity

After 24 h of swelling, four 8 mm discs were cored from each hydrogel containing the peptides and eight were cored from the control. Each disc was transferred to a non-treated cell culture 48-well plate (Falcon, NC, USA) and 100 µL of a solution containing 10 μg/mL of each human GF (PeproTech, NJ, USA) in DPBS was added to the corresponding hydrogel discs and negative controls. The GF solution was left in contact with the hydrogel discs for 1 h at 37 °C and rinsed twice with DPBS. After removing the DPBS, the physically bound GFs to the hydrogels were extracted using 100 µL of lysis buffer (Ambion, Life Technologies, Carlsbad, CA, USA). The lysis solution was in contact with the hydrogel discs for 1 h at room temperature. The supernatant was collected and stored at −20 °C until further use. 

The CBQCA protein quantitation assay (Invitrogen, Life Technologies) was used to quantify the amount of GF bound to the surface of the PEGDA-Peptide hydrogel samples. CBQCA was performed following the manufacturer’s protocol. The growth factors hTGFβ1 and hVEGF were used to build the standard curves for the CBQCA assay. The amount of physically bound peptide to the hydrogel was calculated by interpolation of the fluoresce reading of each sample within the appropriate standard curve and normalized by the total hydrogel surface area. The non-specific absorption of GFs by pure PEGDA (control group) was subtracted from the sample readings from each experimental group. A total of four independent specimens per experimental group were evaluated.

### 5.6. Human Dermal Fibroblasts Cell Culture and Adherence to Hydrogels

Human Dermal Fibroblasts (HDFa, ATCC, CA, USA) at passage 2 were thawed and expanded at 37 °C and 5% CO_2_ using Growth Media (GM, Fibroblast Basal Medium, ATCC) supplemented with Fibroblast Growth kit—low serum (ATCC, 2% Fetal Bovine serum, 5 ng/mL rhFGFβ, 7.5 mM L-glutamine, 50 µg/mL Ascorbic acid, 1 µg/mL Hydrocortisone Hemisuccinate and 5 µg/mL rh-Insulin). 

PEGDA hydrogels containing the KGLPLGNSH or DRVQRQTTTVVA GF biding sequence with 1 mM ACRL-PEG-RGDS were made in sterile conditions, as described in Section 5.3. ACRL-PEG-RGDS was incorporated into the hydrogels to promote cell attachment. An additional hydrogel formulation containing only ACRL-PEG-RGDS was used as the control. Once the gels were constructed, and allowed to swell for 24 h, they were cut using an 8 mm sterile biopsy punch and placed in a non-treated cell culture 48-well plate with DPBS. The DPBS was then removed after 1 h and the corresponding GF solutions, containing 10 ug/mL hTGFβ1, hVEGF or DPBS were added to the corresponding hydrogel formulations. The solutions were in contact with the hydrogels for 1 h and then gently removed. The hydrogels were washed twice with cell culture media. HDFa were harvested at passage 4 and subsequently seeded onto the hydrogel discs at 2000 cell/cm^2^. A portion of the harvested HDFa population was seeded on a cell culture-treated 48-well plate to serve as a 0-day control. Hydrogels were cultured in GM at 37 °C and 5% CO_2_ with a media change every other day.

To evaluate the effects of the bound GFs to the PEGDA-Peptide hydrogel surfaces on cell proliferation, samples were collected (n = 3) at day 3. HDFa cells were fixed with formalin and stored at 4 °C. To assess the effects of physically bound GFs on HDFa phenotype, cells from each hydrogel group (n = 4) were cultured for 5 days. Approximately 125 µL of lysis buffer (Ambion, Life Technologies) was added to each well and incubated at room temperature for 10 min. Following the incubation period, the supernatant was collected and stored at −80 °C for gene expression analysis.

### 5.7. HDFa Proliferation, Surface Coverage, and Average Cell Area

Formalin-fixed cells were rinsed with DPBS and stained with DAPI dilactate (4′,6-diamidino-2-phenylindole, Life technologies, 300 nM) and Rhodamine Phalloidin (Life technologies, 1:100). Between 29 and 36 images were acquired from randomly selected regions of 3 different specimens per experimental group using a Nikon A1 confocal microscope system equipped with a 10× objective. The confocal images were used to quantify the total number of cells per area (number of cells/mm^2^) and the average cell area [64]. The total number of cells was obtained using ImageJ software by counting the number of cell nuclei stained with DAPI. The fraction of area covered by the cells in each image was calculated by first measuring the area in pixels of the cells stained with Rhodamine Phalloidin (Red) using Adobe Photoshop [64]. The total red area was determined using color range to select part of the image covered in red color and a histogram to measure the number of pixels (area). The average cell surface area was calculated by dividing the total red area by the number of cells in each image. The fraction of area coverage was calculated as the total red area divided by the total imaged area.

### 5.8. Gene Expression Analysis

The messenger RNA (mRNA) was extracted using Dynabeads mRNA direct kit (Ambion, Life Technologies) as was previously described by Jimenez-Vergara et al. [65]. In brief, the polyA-mRNA in the supernatant collected in Section 5.5 was harvested using 20 µL of Dynabeads oligo (dT)25 magnetic beads. The obtained mRNA beads were washed twice with buffers A and B and then re-suspended in ice-cold, 10 mM Tris-HCl. The mRNA was retrieved from the beads by heating the resulting solution at 80 °C for 2 min. The supernatant was collected and stored at −80 °C until further use.

Relative mRNA levels for the genes Proliferating Cell Nuclear Antigen (PCNA), Collagen Type I Alpha 1 (COL1A1), TGFβ1, Vimentin and Matrix Metalloproteinase-1 (MMP-1) were calculated using a 7500 Real-Time PCR System (Applied Biosystems, MA, USA) and the SuperScript III Platinum One-Step qRT-PCR kit (Invitrogen, Life Technologies). Primer sequences are shown in Table 5. A total of 25 µL per reaction mixture (≈8 ng of polyA-mRNA and 5 µL of 1 mM primer) was used and changes in SYBR Green fluorescence were monitored in each reaction amplification using the ROX dye as the passive reference. For each sample the gene expression was calculated using the ΔΔCt method. Beta Actin (β-actin) was selected as the housekeeping gene and melting temperatures were used to verify the appropriate amplification products for each PCR reaction.

### 5.9. Statistical Analyses

Data results are reported as the mean ± standard deviation. A comparison of sample means was performed using ANOVA followed by Tukey’s post hoc test (IBM SPSS Statistics software, version: 28.0.1.0 (142)). Differences among experimental groups were considered significant for *p* < 0.05.

## Figures and Tables

**Table 1:** Characteristic absorption IR vibration bands for PEG-acrylate and peptide species.

Bond	Wavenumber [cm^−1^]	Vibration Type
CH_2_	2889	Asymmetrical stretching vibrations
C=O	1721	Symmetrical stretching vibrations
C=O	1650	Amide I band vibrations
C=C	1623	Vibrations of the aliphatic double bond
C=O	1520	Amide II band vibrations
C-O	1110	Stretching vibrations
CH_2_=CH	960	Out-of-plane vibrations symmetrical stretching
CH_2_=CH	843	Symmetrical stretching vibrations

**Table 2:** Physical Characteristics of 6.0 kDa PEGDA-Based Hydrogels.

Concentration [% *w*/*w*]	Complex Modulus (E*) [kPa]	Stiffness [kN/m]	q	q′	M_c_ [kDa/mol]	Mesh Size (ξ) [nm]
10	47.7 ± 7.5	1.9 ± 0.3	15.5 ± 0.7	10.9 ± 0.4	17.5 ± 1.0	21.9 ± 1.0

q is the mass swelling ratio at equilibrium, q′ is the mass swelling ratio at the relaxed state before swelling, and M_c_ is the average molecular weight between crosslinks. *n* = 4.

**Table 3:** Growth Factor Binding Levels on PEGDA-Peptide Hydrogels.

Growth Factor (GF)	Initial GF Concentration in Solution [µg/mL]	GF Bound to PEGDA-Peptides Hydrogels [µg/mL]	Binding Level [ng/mm^2^]
hTGFβ1	10	3.80 ± 0.41	3.19 ± 0.34
hVEGF	10	0.73 ± 0.07	0.62 ± 0.06

A total of 4 independent specimens per experimental group were analyzed.

**Table 4:** PEGDA Binding Peptide (bp) Hydrogel Compositions.

Hydrogel	RGDS [mM]	KGLPLGNSH [mM]	DRVQRQTTTVVA [mM]
Control	1	0	0
hTGFβ1bp	1	1	0
hVEGFbp	1	0	1

All hydrogel precursor solutions contain 10% *w*/*w* 6.0 kDa PEGDA and 1 mM LAP.

**Table 5:** Primers used for qRT-PCR.

Gene	Primer Sequence	Brand
β-actin	F: CACCATTGGCAATGAGCGGTTC	Fisher-Eurofins
	R: AGGTCTTTGCGGATGTCCACGT	
PCNA	F: GCTCCAGCGGTGTAAACCTGCA	Fisher-Eurofins
	R: CGTGCAAAT TCACCAGAAGGCA	
COL1A1	F: GATTCCCTGGACCTAAAGGTGC	Fisher-Eurofins
	R: AGCCTCTCCATCTTTGCCAGCA	
TGFβ1	F: TACCTGAACCCGTGTTGCTCTC	Fisher-Eurofins
	R: GTTGCTGAGGTATCGCCAGGAA	
Vimentin	F: ACGTCTTGACCTTGAACGCA	Fisher-Eurofins
	R: GGCTGCCTTACCCTCATTCA	
MMP-1	F: ATGAAGCAGCCCAGATGTGGAG	Fisher-Eurofins
	R: TGGTCCACATCTGCTCTTGGCA	

## Data Availability

Not applicable.
